# RETRACTION: Chlorpyrifos Suppresses Neutrophil Extracellular Traps in Carp by Promoting Necroptosis and Inhibiting Respiratory Burst Caused by the PKC/MAPK Pathway

**DOI:** 10.1155/omcl/9859263

**Published:** 2026-06-02

**Authors:** Oxidative Medicine and Cellular Longevity

RETRACTION: Q. Zhang, S. Wang, S. Zheng, Z. Zhang, and S. Xu, “Chlorpyrifos Suppresses Neutrophil Extracellular Traps in Carp by Promoting Necroptosis and Inhibiting Respiratory Burst Caused by the PKC/MAPK Pathway,” Oxidative Medicine and Cellular Longevity (2019): 1‐11, https://doi.org/10.1155/2019/1763589.

The above article, published online on 7th February 2019 in Wiley Online Library (wileyonlinelibrary.com), has been retracted by agreement between Editor in Chief Jeannette Vasquez‐Vivar; and John Wiley & Sons Ltd. The retraction has been agreed due to figure concerns initially raised on PubPeer [1]. Specifically, the MAPK3 panel in Figure 3bA is duplicated, flipped horizontally 180 degrees, and stretched from figure 5a in another article by different authors [2]. When the publisher requested the original data and an explanation from the authors, the authors stated that they could not provide their raw data due to a broken hard drive. Because their explanation did not sufficiently answer the concerns of the editors, the results are therefore considered unreliable, and the article is retracted.

The authors disagree with the retraction.
